# Cross-cultural adaptation and content validity of the PGSI into Brazilian Portuguese

**DOI:** 10.11606/s1518-8787.2026060007368

**Published:** 2026-07-17

**Authors:** Carla Cavalheiro Moura, Daniel Tornaim Spritzer, Rodrigo Menezes Machado, Rodrigo Nogueira Borghi, Nathalia Seminário Gabioneta, André Monezi de Andrade, Hermano Tavares

**Affiliations:** I Universidade de São Paulo. Instituto de Psiquiatria. São Paulo, SP, Brazil; II Universidade Federal do Rio Grande do Sul. Programa de Pós-Graduação em Psiquiatria e Ciências do Comportamento. Porto Alegre, RS, Brazil; III Pontifícia Universidade Católica de Campinas. Centro de Ciências da Vida. Campinas, SP, Brazil; IV Universidade São Leopoldo Mandic. Araras, SP, Brazil

**Keywords:** Gambling, Psychometrics, Cross-Cultural Comparison, Brazil, Translations, Behavioral Addictions

## Abstract

**OBJECTIVE:**

This study aimed to cross-culturally adapt the Problem Gambling Severity Index (PGSI) into Brazilian Portuguese and to conduct a preliminary assessment of its content and face validity.

**METHODS:**

The adaptation process followed internationally recognized guidelines and included several stages: initial translation, translation synthesis, back-translation, review by a committee of experts, evaluation by an expert panel of 11 specialists, pre-test with participants from the general population, and final revision. The Content Validity Index (CVI), Clarity Index (CI), and Content Validity Ratio (CVR) were calculated based on the experts’ evaluations. As a first step, establishing semantic and content validity is essential before broader psychometric testing can be meaningfully conducted in the target population.

**RESULTS:**

The adapted PGSI demonstrated strong content validity, with all items reaching a CVI of 0.90 or higher and an overall mean of 0.98. The CI ranged from 2.63 to 2.90, and the CVR indicated high agreement regarding the essentiality of the items. Face validity testing with 33 participants from the general population revealed that all items were rated as understandable or entirely understandable by most participants (84.9% to 100%). Expert feedback was also used to refine the wording of items, particularly to distinguish between “gaming” and “gambling” in the Brazilian context.

**CONCLUSION:**

The Brazilian Portuguese version of the PGSI presented robust preliminary evidence of semantic, conceptual, and cultural adequacy. This version fills a critical methodological gap and provides a culturally appropriate screening and monitoring tool for use in clinical and research settings in Brazil, supporting prevention and public health monitoring of gambling-related harms, including financial impacts. Future studies will evaluate additional psychometric properties further to consolidate its use in clinical and epidemiological contexts.

## INTRODUCTION

Gambling disorder (GD) is defined as a problematic, persistent, and recurrent gambling behavior that leads to clinically significant impairment or distress^1^. Previously termed pathological gambling in the third and fourth editions of the Diagnostic and Statistical Manual of Mental Disorders (DSM) and in the International Classification of Diseases (ICD-10), GD was reclassified from the section “Impulse Control Disorders” to the recently expanded section “Substance-Related and Addictive Disorders” in both DSM-5 and ICD-11^2^. A recent meta-analysis shows that, across five continents, 8.7% of adults were classified as engaging in any risk gambling, and 1.41% were engaged in GD^3^. In the Brazilian context, the lifetime prevalence of problem and pathological gambling was estimated at 2.3% in adults^4^ and 1.6% in adolescents^5^. However, it is important to note that these estimates, collected almost two decades ago, reflect a historical period before the recent legalization of gambling in Brazil and the widespread accessibility of online casinos and betting platforms via smartphones.

The availability of culturally adapted and validated instruments has the potential to support and strengthen national strategies for the prevention and treatment of gambling-related problems^6^. In clinical practice, it can support protocols for early detection, risk stratification, and referral in both primary care and specialized mental health services^7^. From a public health perspective, the instrument can be used to monitor trends in gambling behavior, guide awareness campaigns, and support regulatory efforts amid the growing expansion of online betting in Brazil^8^. Furthermore, it provides a standardized tool for future population surveys and multicenter studies, enabling cross-national comparisons and evidence-based policy formulation.

Several instruments have been used internationally to assess gambling-related problems, including DSM-based diagnostic criteria and DSM-based screening approaches^9^, screening measures originally disseminated in more clinical contexts such as the South Oaks Gambling Screen (SOGS)^10^, instruments derived from diagnostic criteria such as the NORC DSM Screen for Gambling Problems (NODS)^11^, and structured/validated screening measures such as the Gambling Behavior Questionnaire/SOGS-Revised^12^, as well as the Canadian Problem Gambling Index^13^, whose scored component is the Problem Gambling Severity Index (PGSI)^13^. In addition, symptom severity measures frequently used in clinical and treatment research include the Yale–Brown Obsessive Compulsive Scale adapted for pathological gambling^14^ and the Gambling Symptom Assessment Scale^15^.

These approaches are valuable for identifying more severe cases, but they may present limitations for population surveillance when the objective is to capture a continuum of severity and harms (e.g., subclinical levels) and enable comparisons across studies and countries^13^. Among available instruments, the PGSI stands out as a widely used tool to measure the severity of gambling-related problems^16^. The PGSI captures different dimensions of problem gambling, including betting beyond one’s means, tolerance, chasing losses, borrowing money, perceived gambling-related problems, guilt, and health and financial issues^17^. In this context, we justify the choice of the PGSI on methodological, theoretical, and empirical grounds: (i) methodologically, the PGSI was designed for general population surveys, using a 4-point ordinal response format and a continuous score that supports graded risk stratification rather than a strictly dichotomous definition^13^; (ii) theoretically, it aligns with a public health perspective by combining behavioral indicators with adverse consequences/harms associated with gambling; and (iii) empirically, it has undergone extensive psychometric evaluation and is considered a reliable and valid measure^16,18^, with empirical evidence supporting its construct validity, concurrent validity, and sensitivity in assessing symptom severity along a continuum, making it suitable for both clinical use and research with diverse populations^18,19^. Its widespread adoption in population research and psychometric evaluation across settings, including cross-cultural validation across countries^13,18,20,21^, supports international comparability without relying exclusively on diagnostic criteria^13^.

Despite the extensive international use of the PGSI, no Brazilian Portuguese version was available, limiting its application in national research, clinical screening, and cross-national comparisons. By conducting the cross-cultural adaptation of the PGSI into Brazilian Portuguese through a systematic process and presenting preliminary evidence of semantic/cultural adequacy as well as content and face validity, the present study addresses a methodological gap that has limited monitoring and research in Brazil and provides the necessary foundation for subsequent investigations of structural validity, reliability, and other psychometric properties (e.g., factor structure and criterion validity) in larger and more heterogeneous Brazilian samples.

## METHODS

### Cultural Adaptation

The PGSI is a nine-item scale developed to assess the severity of gambling-related problems^13,22^. Response options follow a Likert-type format, ranging from 0 (never) to 3 (almost always). Final scores range from 0 to 27, classifying respondents into four categories: non-gamblers/non-problem gamblers (score 0), low-risk gamblers (scores 1–2), moderate-risk gamblers (scores 3–7), and problem gamblers (scores 8 or higher)^23^. Alternative approaches to categorization and threshold selection have been proposed. Currie et al.^22^ suggested reorganizing the intermediate categories into 1–4 (low risk) and 5–7 (moderate risk) to improve the psychometric coherence of these groups. In addition, population-research classification studies have discussed the potential utility of a ≥ 5 threshold, depending on study aims and context^24^. These findings underscore that PGSI categorization may vary across studies and contexts and that threshold selection can influence prevalence estimates and risk segmentation.

The cross-cultural adaptation process for the Brazilian context followed the guidelines proposed by Sousa and Rojjanasrirat^25^, Beaton et al.^26^, and Polit and Beck^27^. After obtaining consent and authorization from the original developers of the instrument, several stages were conducted, as described below and illustrated in Figure. We based the cross-cultural adaptation workflow on established staged frameworks^26,28,29^. In the present study, the forward translations (T1 and T2) were reconciled into a synthesized version (T1-2), followed by two independent back-translations (BT1 and BT2). Rather than treating BT1–BT2 comparison as a standalone stage, we examined both back-translations within the multidisciplinary committee review, jointly considering all available versions (T1, T2, T1-2, BT1, and BT2) to document and resolve semantic, idiomatic, experiential, and conceptual equivalence and to reach consensus on the final wording.

**Figure:**
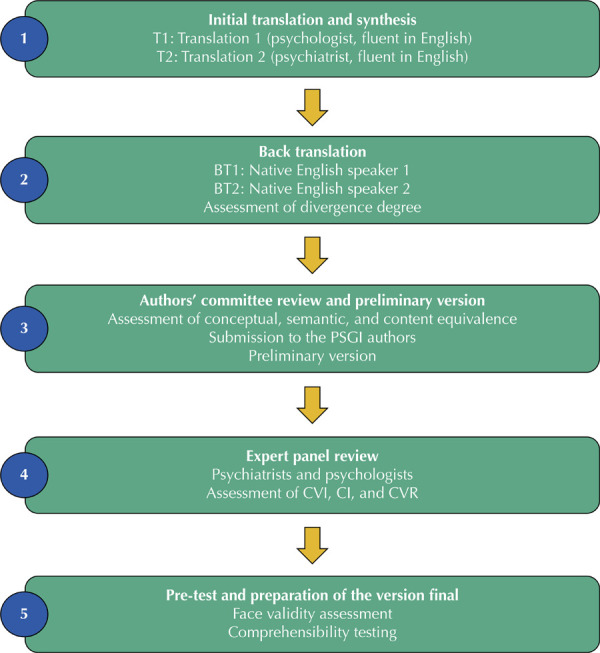
PGSI: Cross-Cultural Adaptation Process, Brazilian Portuguese version.

PGSI: Problem Gambling Severity Index; CVI: Content Validity Index; CI: Clarity Index; CVR: Content Validity Ratio.

Note: cross-cultural adaptation following internationally recognized guidelines^25–27^.

#### Step 1) Initial Translation and Synthesis

The direct translation of the original scale into Brazilian Portuguese (T1 and T2) was performed by two pairs of native Portuguese-speaking, English-fluent specialists in mental health (psychologists and psychiatrists). The two translated versions were comparatively analyzed, and their items were combined into a synthesized version, selecting the best cultural and semantic equivalences. The team of authors performed the synthesis, all of whom are proficient in English and experienced in psychometric assessment. When discrepancies occurred between T1 and T2, reconciliation decisions were made by consensus within the study team, prioritizing conceptual and semantic equivalence to the original English item, clarity and naturalness in Brazilian Portuguese, and consistency with the instrument structure (12-month timeframe and response options). The forward translators subsequently reviewed the synthesized version and agreed with the reconciled wording before the back-translation stage.

#### Step 2) Back-Translation

Two native English speakers independently back-translated the synthesized Brazilian Portuguese version into American English. The back-translations were then evaluated by the study authors. To support the review of the back-translations, the authors developed an item-level divergence rating scale to quantify how different each back-translated item was from the original English version, focusing primarily on meaning. Each item was classified as: unchanged (90% to 100%), slightly changed (70% to < 90%), highly changed (50% to < 70%), or completely changed (< 50%).

#### Step 3) Authors’ Committee Review and Preliminary Version

The two back-translated versions were examined by a committee composed of the study’s authors. They assessed the conceptual, semantic, and content equivalence of the two back-translations until reaching consensus. The finalized back-translation synthesis was submitted to the PGSI developers for validation of the process. Findings from the back-translation were incorporated into the translation synthesis, leading to the development of the preliminary version.

#### Step 4) Expert Panel Review

The preliminary version was submitted to a panel of 11 experts, including researchers and mental health professionals (psychiatrists and psychologists with experience in behavioral addictions and psychometrics). This stage aimed to assess the scale’s content validity and produce a version to be subsequently tested with the general population. The questions addressed the entire translation process, from the instrument’s name and instructions to its item statements and response options. Panelists received each item of the scale in English, along with its Brazilian Portuguese translation, and were asked to evaluate three psychometric indicators of content validity: Content Validity Index (CVI), Clarity Index (CI), and Content Validity Ratio (CVR). The validity indices obtained from these evaluations are presented and detailed below.

## Content Validity Indices

Three key indicators were used to validate the content of the PGSI: CVI, CI, and CVR. The CVI assesses item relevance — whether each item is pertinent to the construct being measured. The CI evaluates the clarity of the language used and its comprehensibility for the target population. The CVR estimates the essentiality of each item, identifying whether its inclusion is fundamental to the instrument’s completeness. The methodology used to calculate these indices followed the guidelines of Polit and Beck^27^, ensuring that the translated scale was appropriate to the cultural and linguistic context of the target population.

The CVI is based on evaluations by experts who assess the relevance of the items, considering cultural and conceptual aspects^30^. Two measures are calculated: the item-level CVI (I-CVI), which reflects the proportion of experts who rate a given item as relevant or highly relevant, and the scale-level CVI (S-CVI), which is the average of all I-CVIs^31^. Reference values between 0.75 and 0.78 are commonly adopted as adequacy thresholds for I-CVI^32^, ensuring that each item retains meaning and applicability within the instrument^33^. Items were rated by experts using a four-point Likert scale: (1) not relevant/representative; (2) needs major modifications to be relevant/representative; (3) relevant/representative but requires minor modifications; and (4) highly relevant/representative. The CVI was calculated by dividing the number of experts who assigned scores of 3 or 4 (indicating relevance) by the total number of evaluators, following the standard dichotomization approach for I-CVI (i.e., 3–4 = relevant; 1–2 = not relevant)^27^. Although this procedure collapses adjacent ordinal categories, it provides a transparent indicator of expert agreement regarding item relevance; in the present study, it was complemented by clarity ratings (CI) and qualitative feedback to preserve nuance in item appraisal.

The CI specifically evaluates the intelligibility of items, ensuring that wording is understandable to the target population^34^. This analysis allows adjustments to item wording before applying the instrument across different cultural and linguistic contexts, ensuring greater validity and reliability in construct measurement^27^. Experts assessed the clarity of each item by assigning scores on a three-point scale: (1) unclear, (2) clear but requires minor modifications, and (3) very clear. The CI was calculated as the mean score (ranging from 1 to 3) given by panelists for each item (i.e., averaged across panelists), thereby focusing on item quality rather than on panelist performance. We considered CI values ≥ 2.0 indicative of adequate clarity (i.e., at least “clear” on average)^27,35,36^.

The CVR evaluates the essentiality of each item, i.e., its representativeness within the scope of the construct being measured^34^. This index helps identify items whose exclusion could compromise the conceptual comprehensiveness of the scale, guiding content adjustments. Experts rated the items on a three-point Likert scale: (1) unnecessary, (2) useful but not essential, and (3) essential. The CVR was calculated using the formula: CVR = (*ne* – [N/2])/(N/2), where *ne* is the number of experts who rated the item as essential and N is the total number of experts^37^. The minimum acceptable CVR was determined according to published critical values for the number of experts^38^; for a panel of 11 experts, the minimum critical CVR is 0.636, corresponding to at least 9 of 11 experts rating an item as “essential”.

In addition, experts were encouraged to provide qualitative comments and suggestions for each item. This approach not only complements quantitative analyses but also allows for more precise refinements in adapting items to the sociocultural context of the target population. Incorporating qualitative feedback enhanced the scale’s terminological and semantic adequacy, ensuring its validity and applicability. Thus, the contributions of experts were crucial to the cultural validation process, ensuring that the final version of the instrument was clear, relevant, and accurately represented its original purpose.

### Step 5) Pre-test and Preparation of the Final Version

At the end of the previous stage, the translated instrument, now referred to as the pilot version, was finalized. A total of 33 individuals completed the translated PGSI between April 10 and 24, 2023. Participants were recruited through convenience sampling in the general population. Inclusion criteria were age ≥ 18 years and the ability to read Brazilian Portuguese. The aim was not to assess the severity of gambling problems in this sample but rather to evaluate whether the cultural adaptation process ensured clarity in the instructions and items in linguistic terms.

## Ethical Considerations

Participants were invited to voluntarily participate in the study after receiving detailed information about its objectives, procedures, and potential risks. The study adhered to the ethical principles outlined in the Declaration of Helsinki. This study was approved by the Research Ethics Committee of the Instituto de Psiquiatria da Universidade de São Paulo (CAAE: 12820813.5.0000.0068).

## RESULTS

### Cultural Adaptation

This Chart compares the original English version and the final translated version of the PGSI. The final version resulted from several stages of the cross-cultural adaptation process, including translation and synthesis, back-translation, committee review, expert panel evaluation, and pre-testing with participants from the general population. The adaptations aimed to preserve the semantic, conceptual, and idiomatic equivalence of the items, ensuring clarity, relevance, and cultural appropriateness in the Brazilian context.

**Chart:** Comparison between the original and final Brazilian Portuguese version of the PGSI.

Original version	Final version
Problem Gambling Severity Index	*Índice de gravidade de problemas com apostas*
Thinking about the last 12 months...	*Pensando nos últimos 12 meses...*
Never | sometimes | most of the time | almost always	*Nunca | às vezes | na maioria das vezes | quase sempre*
Have you bet more than you could really afford to lose?	*Você apostou mais do que realmente poderia perder?*
Still thinking about the last 12 months, have you needed to gamble with larger amounts of money to get the same feeling of excitement?	*Ainda pensando nos últimos 12 meses, você precisou apostar quantias cada vez maiores de dinheiro para ter a mesma sensação de prazer?*
When you gambled, did you go back another day to try to win back the money you lost?	*Depois de ter apostado, você retorna outro dia para tentar recuperar o dinheiro perdido?*
Have you borrowed money or sold anything to get money to gamble?	*Você pediu dinheiro emprestado ou vendeu alguma coisa para conseguir dinheiro para apostar?*
Have you felt that you might have a problem with gambling?	*Você achou que poderia ter algum problema com apostas?*
Has gambling caused you any health problems, including stress or anxiety?	*Apostar já lhe causou algum problema de saúde, como estresse ou ansiedade?*
Have people criticized your betting or told you that you had a gambling problem, regardless of whether or not you thought it was true?	*As pessoas já lhe criticaram por apostar, ou disseram que você tinha problemas com apostar, independentemente de você achar que era verdade ou não?*
Has your gambling caused any financial problems for you or your household?	*As suas apostas já causaram algum problema financeiro para você ou sua família?*
Have you felt guilty about the way you gamble or what happens when you gamble?	*Você já se sentiu culpado(a) pela maneira como você aposta ou pelo que acontece quando você aposta?*

PGSI: Problem Gambling Severity Index.

### Content Validity

Table 1 shows that the content validity assessment of the Brazilian Portuguese version of the PGSI demonstrated high levels of agreement among experts. The overall CVI was greater than 0.90, indicating that most items were considered clear and relevant. The CI also showed satisfactory values, ranging from 2.63 to 2.90, reinforcing the adequacy of the translated items. In addition, the CVR indicated that the evaluators widely recognized the essentiality of the items.

**Table 1:** Content validity index results of the PGSI Brazilian Portuguese version.

Item	CVI	CI	CVR
1	1.00	2.63	1.00
2	1.00	2.90	1.00
3	1.00	2.81	1.00
4	1.00	2.90	1.00
5	0.90	2.63	1.00
6	1.00	2.81	0.91
7	1.00	2.72	1.00
8	1.00	2.81	1.00
9	1.00	2.72	1.00
S-CVI	0.98	-	-

PGSI: Problem Gambling Severity Index; CVI: Content Validity Index; CI: Clarity Index; CVR: Content Validity Ratio; S-CVI: Scale-level Content Validity Index.

Notes: CVI: proportion of experts who classified the item as relevant or highly relevant (scores 3 or 4). Values ≥ 0.78 are considered adequate (Polit and Beck, 2006).

CI: average of scores attributed by experts about the clarity of each item, on a scale from 1 (unclear) to 3 (very clear). Values ≥2.0 indicate adequate clarity.

CVR: calculates the essentiality of each item using the formula CVR = (ne – [N/2]) / (N/2), where ne = number of experts who classified the item as essential and n = total number of experts (11). Values ≥ 0.63 are considered adequate for 11 experts^38^.

S-CVI: average of CVIs of all items, representing the general content validity of the scale.

Item 5 showed comparatively lower agreement, and expert feedback indicated that this was mainly due to conceptual/semantic equivalence in Brazilian Portuguese (i.e., how to best express subjective awareness of a potential gambling-related problem). Panelists proposed alternative formulations (including wording closer to “feeling” or “thinking recently” about having a problem) and highlighted potential ambiguity in the phrasing, which likely contributed to its lower index compared with the remaining items.

Throughout the cross-cultural adaptation process, some experts provided specific comments and suggestions regarding the translation of the scale, which were duly documented. The main observations focused on the choice of terminology (“*apostas” versus* “*jogo”*), highlighting the importance of terminological precision in preserving the conceptual meaning of the items. In the Brazilian context, the word “*jogo”* is widely used to refer both to gambling and video gaming. To preserve clarity and avoid terminological ambiguity, the term *“apostas”* was chosen for the translated version, replacing the more general term “*jogo”*.

### Face Validity

Table 2 presents the results of the face validity assessment, conducted with 33 participants from the general population. The PGSI scale items were rated as understandable or fully understandable by most respondents, with rates ranging from 84.9% to 100%.

**Table 2:** Comprehension of PGSI scale items by participants from the general population (n = 33).

Face validity			
Item	Totally understandable	Understandable	Nothing or almost nothing is understandable
1	84.9%	12.1%	3.0%
2	93.9%	6.1%	0%
3	93.9%	6.1%	0%
4	100%	0%	0%
5	100%	0%	0%
6	100%	0%	0%
7	100%	0%	0%
8	100%	0%	0%
9	94.0%	3.0%	3.0%

PGSI: Problem Gambling Severity Index.

## DISCUSSION

The present study aimed to cross-culturally adapt and assess the content validity of the Brazilian version of the PGSI. The results provided robust evidence of semantic and content validity: all items demonstrated a CVI ≥ 0.90, with an overall mean of 0.98 — a value well above the cutoff point of 0.78 recommended for instruments undergoing validation^27^ and consistent with references used in national and international studies. The CI, with values ranging from 2.63 to 2.90, and the CVR, ranging from 0.91 to 1, further reinforce the linguistic and conceptual adequacy of the scale for the Brazilian context. Although content validity indices were high overall, with all items exceeding 0.90, minor variations were observed among items. Some experts raised specific comments on terminology and item clarity, particularly regarding colloquial usage or regional interpretations.

The adoption of widely recognized methodological guidelines ensured semantic, idiomatic, conceptual, and cultural equivalence in the adapted version^25^. This approach is considered essential for establishing content validity in psychometric instruments. Most items remained conceptually unchanged, preserving their original structure and intent. However, specific modifications were made to address cultural nuances. For example, the term “gambling” was translated as “*apostas*” rather than “*jogo*”, because “*jogo*” is used more broadly and can be ambiguous in Brazilian Portuguese, where it also refers to video games. This change was essential to avoid confusion and accurately capture the construct assessed by the PGSI.

Expert suggestions were carefully considered and, when appropriate, incorporated into the final version to improve clarity and cultural alignment. Importantly, when expert ratings deviated from full endorsement, qualitative feedback indicated that this typically reflected refinement needs rather than substantive disagreement with item relevance. Deviations often reflected three main concerns: first, semantic nuance and conceptual equivalence, particularly for items requiring natural, non-stigmatizing phrasing in Brazilian Portuguese (e.g., Item 5, where experts debated whether wording such as “*sentiu*” *versus* “*achou*” better captured the intended meaning); second, temporal and frequency framing to avoid interpreting behaviors as single past events, leading to suggestions to adjust verb tense and sequencing language (e.g., Items 1 and 3) to reinforce that items refer to experiences within the last 12 months; and third, potential minimization or resistance in self-report formats for items assessing consequences (e.g., Item 8), underscoring the need for clear, culturally natural wording. Consistent with this interpretive rationale, minor wording refinements were implemented between the expert panel stage (Step 4) and the pre-test (Step 5) (e.g., removal of “já” in Item 1), while preserving the 12-month timeframe and the response format. Although no item was rated as irrelevant or inadequate, expert feedback was valuable for refining the instrument and ensuring that each item maintained conceptual fidelity while remaining accessible to the target population.

The content validity results obtained for the Brazilian Portuguese version of the PGSI are broadly in line with international experiences of cultural adaptation, but with some methodological distinctions. Most validations in other contexts, such as the Chinese^18^ and Spanish^19^ versions, followed the classical steps of translation, back-translation, expert review, and pre-test, ensuring semantic equivalence but without reporting formal indices of content validity. The Japanese study added the contribution of the original authors of the PGSI to confirm semantic equivalence^21^. At the same time, the Persian version^20^ adhered strictly to the guidelines of Beaton et al.^26^ and was complemented by a pilot test, though, again, without a systematic quantitative evaluation of content validity. In contrast, the Brazilian process combined these traditional steps with multiple quantitative indicators (CVI, CI, and CVR), which, together with qualitative feedback, enabled a more detailed appraisal of item clarity, relevance, and essentiality.

The use of expert panels composed of mental health professionals familiar with GD represents a methodological strength that enhances the credibility of the content validity results. This approach ensures that the cultural adaptation process benefits from clinical and research expertise specific to the Brazilian context while maintaining fidelity to the theoretical framework of the original instrument. The face validity assessment with participants from the general population (comprehensibility rates of 84.9% to 100%) provides further evidence of the instrument’s accessibility and clarity in the Brazilian context. This step, often overlooked in validation studies, ensures that the adapted instrument is not only validated by experts but also understandable to its intended users. Overall, this approach ensured semantic and cultural adequacy for the Brazilian context and offers a methodological contribution by demonstrating how systematic content validity analyses can complement traditional adaptation procedures in future cross-cultural validations.

The Brazilian version of the PGSI emerges as a promising tool for use in various contexts, from specialized services and primary care to school-based prevention programs and multicenter studies. Its simple structure, transparent and accessible language, and risk-level categorization facilitate its application in both screening and research settings. Previous studies have shown that the PGSI is more sensitive than other instruments in identifying moderate-risk behaviors in non-clinical populations^39^. Furthermore, the PGSI has been recommended for use in national mental health surveys due to its ability to assess gambling behavior along a continuum of severity^13^.

This development comes at a critical moment, marked by the growing liberalization of the gambling market and the increasing popularity of online sports betting in Brazil, particularly among young people and adults^40^. Prevalence estimates of GD may increase in future studies due to factors such as greater gambling availability, greater social acceptance, and the ongoing legalization process in Brazil^41^. From a public health perspective, gambling-related harms are not distributed evenly across the population. Evidence from Brazil suggests that vulnerability to gambling problems is associated with markers of social disadvantage and poor social insertion, including unemployment and not being enrolled in education, and that the risk is higher among individuals from lower socioeconomic backgrounds^4,5,41^. An economic perspective also underscores the relevance of early detection: self-reported gambling expenditure has been shown to represent a substantial share of household income among individuals with more severe gambling problems, indicating disproportionate financial strain at higher severity levels. Moreover, specialized services are limited and unevenly distributed, with lower socioeconomic regions, where risk may be higher, being less covered by specialized care, which can amplify inequities in access to treatment and prevention^41,42^. In this context, the availability of a culturally adapted PGSI can support more equitable surveillance and screening efforts, helping to identify at-risk groups and inform targeted prevention, regulation, and referral strategies.

Despite this rigorous adaptation methodology and positive findings, this study presents some limitations that should be acknowledged. It focused on assessing content and face validity, without addressing additional psychometric properties, such as factor structure, internal consistency, test-retest reliability, or criterion validity. These characteristics are essential to ensuring the validity of the instrument in both clinical and research settings. Additionally, although face validity testing indicated high comprehensibility rates, the pre-test was conducted with a relatively small convenience sample. This limitation may restrict the extent to which the findings capture the linguistic and cultural heterogeneity of the Brazilian population, which is characterized by substantial regional and sociocultural diversity. Accordingly, subsequent studies with larger and more heterogeneous samples are warranted to evaluate structural validity, reliability (including test–retest), and criterion-related validity in both clinical and population-based settings. Future investigations will be necessary to evaluate these dimensions and establish the Brazilian version as a fully validated instrument.

The Brazilian Portuguese version of the PGSI demonstrated conceptual equivalence and cultural adequacy, with strong preliminary evidence of content and face validity. This adaptation fills an important methodological gap, providing a culturally appropriate tool for screening and research in Brazil. The availability of this version has practical implications for clinical practice, public health strategies, and regulatory policies, as it enables the early identification of gambling-related problems, supports prevention and intervention initiatives, and facilitates cross-national comparisons in a rapidly changing gambling landscape. Further psychometric studies are warranted to consolidate its use, but the present findings position the Brazilian PGSI as a valuable resource for both research and applied contexts.

## Data Availability

The data of the current study are available from the corresponding author on reasonable request.
